# Metastasis pattern and prognosis in men with esophageal cancer patients: A SEER-based study

**DOI:** 10.1097/MD.0000000000026496

**Published:** 2021-06-25

**Authors:** Shengqiang Zhang, Jida Guo, Hongyan Zhang, Huawei Li, Mohamed Osman Omar Hassan, Linyou Zhang

**Affiliations:** a Department of Thoracic Surgery, The Second Affiliated Hospital of Harbin Medical University, Harbin; b Department of Physiology and Neurobiology, Mudanjiang Medical University, Mudanjiang, China; c Department of Cardiothoracic Surgery, Qena University Hospitals, Qena Faculty of Medicine, South Valley University, Qena, Egypt.

**Keywords:** esophageal cancer, male, metastasis, prognosis, SEER

## Abstract

Esophageal cancer (EC) is relatively common; at the time of diagnosis, 50% of cases present with distant metastases, and most patients are men. This study aimed to examine and compare the clinicopathological characteristics and metastatic patterns of male EC (MEC) and female EC (FEC). In addition, risk factors associated with MEC prognosis were evaluated.

The present study population was extracted from the Surveillance Epidemiology and End Results database. MEC characteristics and factors associated with prognosis were evaluated using descriptive analysis, the Kaplan–Meier method, and the Cox regression model.

A total of 12,558 MEC cases were included; among them, 3454 cases had distant organ metastases. Overall, 27.5% of the entire cohort were patients with distant organ metastases. Compared with patients with non-metastatic MEC, patients with metastatic MEC were more likely to be aged ≤60 years, of Black and White race, have a primary lesion in the overlapping esophagus segments, and have a diagnosis of adenocarcinoma of poorly differentiated and undifferentiated grade that was treated with radiotherapy and chemotherapy rather than surgery; moreover, they were also more likely to be married and insured. In addition, patients with MEC were more likely to be aged ≤60 years, White race, and diagnosed with a primary lesion in the lower third of the esophagus and overlapping esophagus segments, and treated without chemotherapy, compared with those with FEC. Patients in the former group were also more likely than those in the latter group to be unmarried and have bone metastasis only and lung metastasis only. Liver, lung, and bone metastases separately, and simultaneous liver and lung metastases were associated with poor survival in MEC patients.

Metastatic MEC is associated with clinicopathological characteristics and metastatic patterns different from those associated with non-metastatic MEC and metastatic FEC. Metastatic MEC and FEC patients may have similar prognoses. Distant organ metastasis may be associated with poor prognosis in patients with MEC and FEC.

## 1 Introduction

Esophageal cancer (EC) is the seventh most common cancer and the sixth leading cause of mortality worldwide.^[1]^ In 2019, 17,650 new EC cases and 16,080 associated deaths were reported in the United States,^[2]^ including 13,750 new diagnoses and 13,020 deaths among men. Men account for two-thirds of EC patients.^[1,3–5]^ Approximately, 50% of EC patients presented with distant site metastases at the time of diagnosis.^[6,7]^

EC survival has improved with progress in treatment. Some population-based studies have reported that the overall 5-year survival rates of this patient group increased from <5% in the 1960s to >20% in the past decade.^[8]^ However, in some European countries, the United States, and China, the prognosis of male EC (MEC) patients remains poor, in particular, in cases of distant organ metastasis.^[9]^ Previous studies have reported on distant metastasis of EC; however, a systematic examination of MEC with distant metastasis has not been undertaken to date; thus, the clinical characteristics, metastatic patterns, and factors associated with prognosis in this patient group remain unclear.

The present study was based on a large population of patients with EC and metastasis. The data were extracted from the Surveillance Epidemiology and End Results (SEER) database (2010–2015). This study aimed to examine the clinical and epidemiological characteristics of MEC. Moreover, EC characteristics were compared between male and female EC (FEC) patients. Factors affecting MEC prognosis were examined.

## 2 Methods

### 2.1 Populations and characteristics

**Figure 1:**
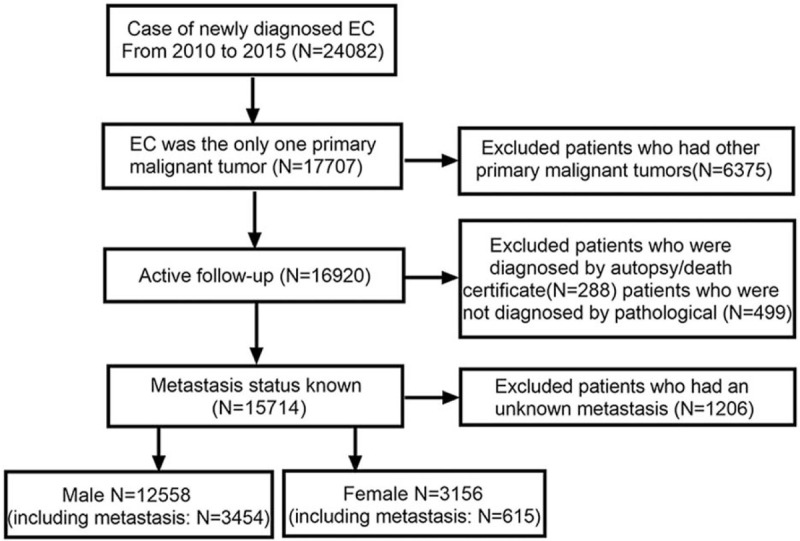
Flowchart of selection of patients with metastatic esophageal cancer used the SEER database. SEER = surveillance epidemiology and end results.

SEER∗stat software was used to extract data from the SEER-18 database, which is maintained by the National Cancer Institute and accounts for approximately 28% of the United States’ population. SEER∗stat provided information up to 2016; we included patients aged ≥18 years diagnosed with EC between January 1, 2010 and December 31, 2015 (N = 24,082). We excluded patients with diagnoses of other primary malignant tumors, diagnoses made at autopsy or via a death certificate, diagnosis not pathologically confirmed, and those who lacked information about distant organ metastasis status (Fig. 1).

Characteristics of interest were age at diagnosis (≤60 years old and >60 years old); race (White, Black, and other race); primary lesion site (esophagus upper1/3, esophagus middle1/3, esophagus lower1/3, and spanning 2 or more esophageal segments means overlapping lesion); histopathology type (squamous cell carcinoma, adenocarcinoma, and other types); pathology grade (I well-differentiated, II moderately, III poorly differentiated, and IV undifferentiated); surgery, radiotherapy, chemotherapy, marital, and insurance status; and residence type.

Distant metastases were observed in the bone, brain, liver, and lungs. Metastasis patterns were divided into 15 groups: single-organ metastases (bone, brain, liver, and lung), 2-organ metastases (bone and brain, bone and liver, bone and lung, brain and liver, brain and lung, and liver and lung), 3-organ metastases (bone, brain, and liver; bone, brain, and lung; bone, liver, and lung; and brain, liver, and lung), and4-organ metastases (bone, brain, liver, and lung).

### 2.2 Statistical analysis

In this cohort study, descriptive analysis was used to calculate the proportion and an absolute number of cases per characteristic. The chi-squared test or Fisher exact test was used for between-group comparisons of characteristics. The metastatic proportion was defined as the percentage of patients with MEC with or without distant organ metastasis to the total number of MEC cases, and the number of FEC with distant metastasis to the total number of FEC cases. Survival estimates were obtained with the Kaplan–Meier method and compared using the log-rank test in MEC and FEC patients with different metastasis organs; univariable Cox regression was used to determine factors associated with all-cause mortality. Factors statistically significant (*P* < .05) in univariable analysis were entered into the multivariable Cox regression model.

The Kaplan–Meier curves were generated using GraphPad Prism software (version 8.0; La Jolla, CA, USA); other statistical analyses were performed with SPSS (version 25.0; IBM Corp., USA). Statistical significance was set as two-sided *P*-values of <.05. The SEER dataset used was publicly available and thus exempt from an ethics board review and the informed consent requirement. This study complied with the 1964 Helsinki Declaration and its subsequent amendments and other relevant ethical standards.

## 3 Results

### 3.1 Patients’ criteria

Among 12,558 MEC cases, 3454 patients had metastases. Among 3156 FEC cases, 615 patients had metastases. All patients were diagnosed during 2010–2015; the age range was 18 to 105 years. MEC and FEC patients with metastasis were aged 63.68 ± 11.135 and 66.30 ± 12.525 years, respectively. Metastatic MEC patients were more likely than their counterparts to be aged <60 years, of Black or White race, married, and without insurance, and to have an overlapping lesion primary site, and the diagnosis of poorly differentiated (grade III) or undifferentiated (grade IV) adenocarcinoma that was treated with radiotherapy and chemotherapy rather than surgery. The place of residence was similar in both groups. The patients’ clinical and demographic characteristics are presented in Table 1.

**Table 1:** Clinical characteristics of male and female patients with esophageal cancer.

	MEC without metastasis		MEC with metastasis		FEC without metastasis		FEC with metastasis			
	N 9104	% 72.5	N 3454	% 27.5	N 2541	% 80.5	N 615	% 19.5	*P* value^†^	*P* value^‡^
Age at diagnosis(year)									.000^∗∗^	.046^∗^
≤60	2858	31.4	1343	38.9	647	25.5	213	34.6		
>60	6246	68.6	2111	61.1	1894	74.5	402	65.4		
Race									.001^∗^	.000^∗∗^
Black	758	8.3	322	9.3	389	15.3	99	16.1		
White	7792	85.6	2973	86.1	1991	78.4	486	79.0		
Others	512	5.6	154	4.5	149	5.9	29	4.7		
Unknown	42	0.5	5	0.1	12	0.5	1	0.2		
Primary site									.000^∗∗^	.000^∗∗^
Upper1/3	518	5.7	143	4.1	283	11.1	40	6.5		
Middle1/3	1460	16.0	455	13.2	772	30.4	144	23.4		
Lower1/3	6102	67.0	2266	65.6	1186	46.7	325	52.8		
Overlapping lesion	364	4.0	215	6.2	107	4.2	25	4.1		
Unknown	660	7.2	375	10.9	193	7.6	81	13.2		
Histopathology type									.000^∗∗^	.000^∗∗^
Squamous cell carcinoma	2363	26.0	709	20.5	1381	54.3	230	37.4		
Adenocarcinoma	5938	65.2	2443	70.7	979	38.5	328	53.3		
Others	803	8.8	302	8.7	181	7.1	57	9.3		
Pathology grade									.000^∗∗^	.565
I well-differentiated	540	5.9	86	2.5	145	5.7	14	2.3		
II moderately	3103	34.1	943	27.3	965	38.0	168	27.3		
III poorly differentiated	3682	40.4	1700	49.2	859	33.8	289	47.0		
IV undifferentiated	111	1.2	56	1.6	32	1.3	8	1.3		
Unknown	1668	18.3	669	19.4	540	21.3	136	22.1		
Surgery									.000^∗∗^	.111
No	5578	61.3	3376	97.7	1843	72.5	602	97.9		
Yes	3362	36.9	67	1.9	653	25.7	8	1.3		
Unknown	164	1.8	11	0.3	45	1.8	5	0.8		
Radiotherapy									.000^∗∗^	.562
No	5949	65.3	1340	38.8	1013	39.9	231	37.6		
Yes	3155	34.7	2114	61.2	1528	60.1	384	62.4		
Chemotherapy									.000^∗∗^	.034^∗^
No	6229	68.4	2089	60.5	1049	41.3	344	55.9		
Yes	2875	31.6	1365	39.5	1492	58.7	271	44.1		
Marital status									.000^∗∗^	.000^∗∗^
Married	3249	35.7	1372	39.7	907	35.7	366	59.5		
Unmarried	5323	58.5	1925	55.7	1482	58.3	215	35.0		
Unknown	532	5.8	157	4.5	152	6.0	34	5.5		
Residence type									.270	.378
Rural	173	1.9	53	1.5	33	1.3	5	0.8		
Urban	8911	97.9	3396	98.3	2498	98.3	609	99.0		
Unknown	20	0.2	5	0.1	10	0.4	1	0.2		
Insurance situation									.000^∗∗^	.114
Insurance	8581	94.3	3221	93.3	2399	94.4	562	91.4		
No insurance	310	3.4	168	4.9	77	3.0	34	5.5		
Unknown	213	2.3	65	1.9	65	2.6	19	3.1		

FEC = female esophageal cancer, MEC = male esophageal cancer

† Comparison between male esophageal cancer without metastasis and male esophageal cancer with metastasis.

‡ Comparison between male esophageal cancer with metastasis and female esophageal cancer with metastasis.

∗ *P* < .05.

∗∗ *P* < .001.

MEC patients with distant organ metastasis were more likely than their female counterparts to be aged <60 years, unmarried, of White race, diagnosed with adenocarcinoma in the lower third of the esophagus and overlapping lesions; concurrently, the former group was less likely than the latter group to receive chemotherapy. Pathology grade, surgery and radiotherapy status, residence type, and insurance status were similar in both groups.

### 3.2 Metastasis patterns

There were 3454 patients in this cohort. The most common distant single-, 2-, and 3-organ metastasis sites were the liver (N = 1238, 35.8%), liver and lung (N = 466, 13.5%), and bone, liver, and lung (N = 140, 4.1%), respectively. Overall, the most common metastasis patterns were liver metastasis only (N = 1238, 35.8%), lung metastasis only (N = 483, 14.0%), bone metastasis only (N = 482, 14.0%), and concurrent liver and lung metastasis (N = 466, 13.5%).

MEC patients with distant organ metastasis were more and less likely than their FEC counterparts to have bone metastasis only (14.0% vs 10.9%), and lung metastasis only (14.0% vs 24.6%), respectively. The remaining metastatic patterns were observed at a similar frequency in both groups (Table 2).

**Table 2:** Compare organ metastasis patterns between male and female patients with esophageal cancer.

	Male		Female		
	N = 3454		N = 615		*P* value
Variable	n	%	n	%	
Bone metastasis only	482	14.0	67	10.9	.041^∗^
Brain metastasis only	95	2.8	10	1.6	.105^∗^
Liver metastasis only	1238	35.8	204	33.2	.202^∗^
Lung metastasis only	483	14.0	151	24.6	<.001^∗^
Bone and brain	29	0.8	4	0.7	.809^∗∗^
Bone and liver	268	7.8	40	6.5	.278^∗^
Bone and lung	117	3.4	20	3.3	.864^∗^
Brain and liver	39	1.1	3	0.5	.147^∗^
Brain and lung	20	0.6	6	1.0	.268^∗∗^
Liver and lung	466	13.5	74	12.0	.326^∗^
Bone, brain, and liver	14	0.4	5	0.8	.192^∗∗^
Bone, brain, and lung	11	0.3	5	0.8	.081^∗∗^
Bone, liver, and lung	140	4.1	22	3.6	.578^∗^
Brain, liver, and lung	27	0.8	2	0.3	.300^∗∗^
Bone, brain, liver, and lung	25	0.7	2	0.3	.416^∗∗^
One site metastasis	2298	66.5	432	70.2	.071^∗^
Two sites metastasis	939	27.2	147	23.9	.090^∗^
Three sites metastasis	192	5.6	34	5.5	.976^∗^
Four sites metastasis	25	0.7	2	0.3	.416^∗∗^

∗ Pearson chi-squared test.

∗∗ Fisher exact test.

### 3.3 Survival

The patients with the above-mentioned metastasis patterns accounted for over 80% of the sample and were included in survival analysis.

**Figure 2:**
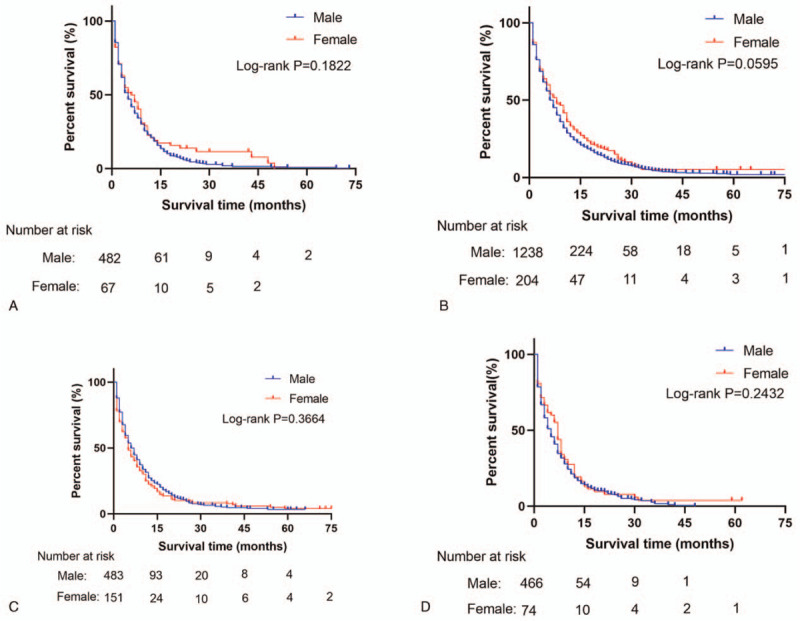
OS rate of MEC and FEC patients at different metastasis sites. (A) OS of bone alone metastasis between MEC and FEC patients; (B) OS of liver alone metastasis between MEC and FEC patients; (C) OS of lung alone metastasis between MEC and FEC patients; and (D) OS of both liver and lung metastasis between MEC and FEC patients. FEC = female esophageal cancer, MEC = male esophageal cancer, OS = overall survival.

**Figure 3:**
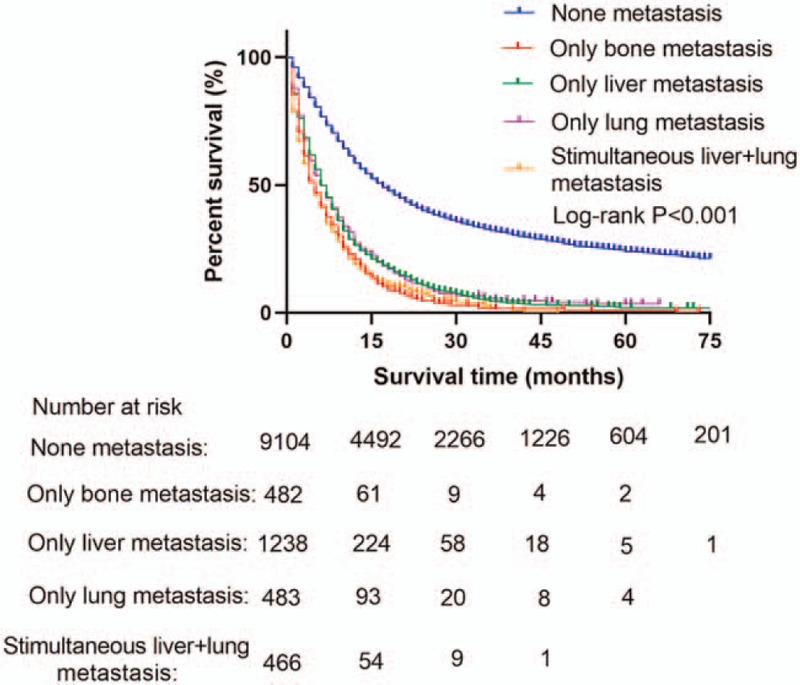
The survival difference among the different metastasis sites in MEC patients. MEC = male esophageal cancer.

**Figure 4:**
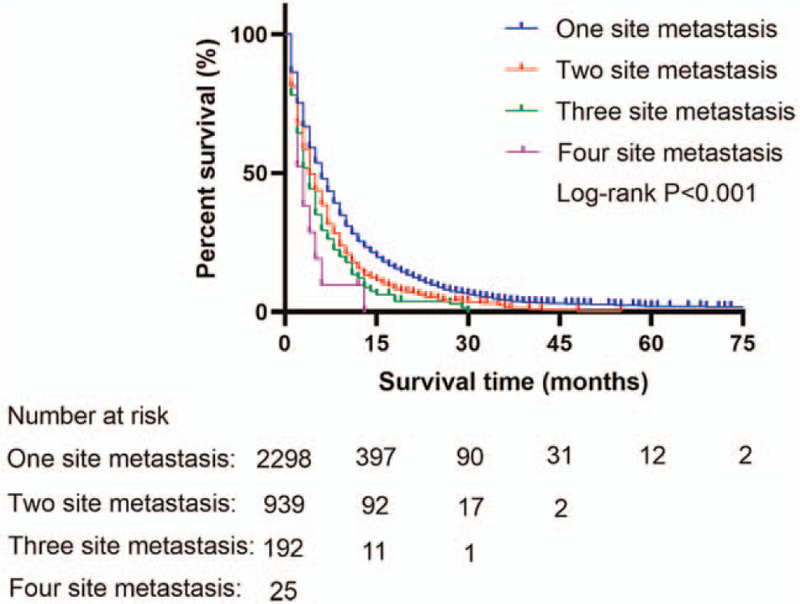
The survival difference among the different number of metastasis sites in MEC patients. MEC = male esophageal cancer

There was no difference in survival rates between metastatic MEC and FEC patients (Fig. 2). However, overall survival rates were different between metastatic and non-metastatic MEC patients (Fig. 3). Among patients with metastatic MEC, survival rates decreased with an increase in the number of metastatic sites (Fig. 4).

Univariate Cox regression revealed 11 factors associated with all-cause mortality in metastatic MEC patients (Table 3). Multivariable Cox regression revealed that the following factors were associated with poor prognosis: age at diagnosis of >60 (vs ≤60) years, primary lesion site in the middle or lower third of the esophagus, and overlapping lesions (vs lesions in the upper third), pathology grade II (moderately differentiated), III (poorly differentiated), and IV (undifferentiated) (vs grade I, well-differentiated), no insurance (vs insurance), and distant organ metastatic (vs non-metastatic). Concurrently, factors associated with good prognosis in this patient group were being of non-Black race, diagnosis of adenocarcinoma (vs squamous cell carcinoma), treatment with surgery (vs without surgery) and chemotherapy (vs without chemotherapy), and being married (vs unmarried). Radiotherapy did not affect outcomes in the present study.

**Table 3:** Univariate and multivariate survival analysis of male esophageal cancer patients with bone alone, liver alone, lung alone, and simultaneous liver and lung metastasis.

	Univariate analysis	Multivariate analysis		
Characteristics	*P*	Hazard ratio	95%CI	*P* value
Age at diagnosis (year)	<.001			.011
≤60		Reference		
>60		1.062	1.014–1.113	.011
Race	<.001			<.001
Black		Reference		
White		0.921	0.852–0.995	.038
Others		0.867	0.775–0.971	.014
Unknown		0.401	0.247–0.652	<.001
Primary site	.010			<.001
Upper1/3		Reference		
Middle1/3		1.178	1.062–1.306	.002
Lower1/3		1.217	1.101–1.346	<.001
Overlapping lesion		1.460	1.280–1.665	<.001
Unknown		1.272	1.129–1.432	<.001
Histopathology type	<.001			<.001
Squamous cell carcinoma		Reference		
Adenocarcinoma		0.844	0.794–0.898	<.001
Others		0.964	0.882–1.053	.414
Pathology grade	<.001			<.001
I well-differentiated		Reference		
II moderately		1.430	1.275–1.605	<.001
III poorly differentiated		1.872	1.670–2.098	<.001
IV undifferentiated		1.932	1.564–2.386	<.001
Unknown		1.295	1.149–1.461	<.001
Surgery	<.001			<.001
No		Reference		
Yes		0.277	0.261–0.295	<.001
Unknown		0.652	0.543–0.782	<.001
Radiotherapy	<.001			.526
No		Reference		
Yes		1.017	0.966–1.070	.526
Chemotherapy	<.001			<.001
No		Reference		
Yes		0.534	0.507–0.562	<.001
Marital status	<.001			<.001
Unmarried		Reference		
Married		0.846	0.809–0.885	<.001
Unknown		0.802	0.725–0.886	<.001
Residence type	.882	NA		
Rural				
Urban				
Unknown				
Insurance situation	<.001			<.001
Insurance		Reference		
No insurance		1.262	1.132–1.406	<.001
Unknown		0.850	0.730–0.989	.035
Metastasis	<.001			<.001
None		Reference		
Bone only		2.105	1.910–2.320	<.001
Liver only		1.836	1.712–1.968	<.001
Lung only		1.609	1.458–1.776	<.001
Liver and lung		2.372	2.146–2.623	<.001

CI = confidence intervals.

## 4 Discussion

In the present study, we examined the clinical characteristics, metastatic patterns, and factors affecting prognosis in patients with metastatic MEC, registered in the SEER database. Outcomes were compared among metastatic and non-metastatic MEC patients and metastatic FEC patients. In the present study, 27.5% of MEC patients were metastatic cases; this rate was higher than that of the FEC group. The clinicopathological characteristics were different between the cohorts. To the best of our knowledge, this is the first large study on EC metastasis in men.

Some previous studies have shown similar findings in patients with metastatic EC,^[7,9]^ including higher incidence among men than among women; in the present study, the rate of EC was 1.5-fold higher among men than among women. Male sex hormones may promote EC cell proliferation and metastasis,^[10,11]^ while men are more likely than women to drink alcohol and smoke cigarettes. These hormonal and behavioral differences between men and women may account for the differences in EC rates between the sexes.

Younger patients with EC are more likely than their older counterparts to have metastasis^[12]^; men are particularly vulnerable to metastatic EC, as seen in the present study. In the populations of the United States and Europe, adenocarcinoma is the main histopathological type of EC,^[1,13]^ particularly common among men with metastatic EC. Poorly differentiated malignant tumors are associated with poor prognosis and increased incidence of distant organ metastasis.^[14,15]^ Advanced-stage cancers tend to be treated with chemotherapy and radiation rather than surgery, which might also be the reason why patients with metastatic MEC rarely receive surgery. Metastatic MEC patients were treated with surgery and radiotherapy at a rate similar to that observed in metastatic FEC patients, and with marginally reduced rates of chemotherapy. Metastatic MEC patients were more and less likely than non-metastatic MEC and FEC patients to be married, respectively. Understanding whether malignant tumor metastasis is associated with marital status requires further research. The rate of insurance holders was lower among patients with metastatic MEC than among those with non-metastatic MEC; this rate was similar to that among metastatic FEC patients. Uninsured patients have reduced access to medical care, which may increase their risk of progressing to advanced-stage cancer.

We compared distant organ metastasis patterns between MEC and FEC patients; overall, the most and least common metastatic sites among EC patients were the liver and brain, respectively; this finding is consistent with that of previous studies.^[16–19]^ The incidence of bone-only metastasis among metastatic MEC patients was higher than that among metastatic FEC patients.^[20]^ Ma et al reported that female sex was associated with a lower risk of bone metastasis in digestive system cancers.^[21]^ Sex hormone levels differ between men and women, and musculoskeletal health may be the primary cause of these differences.^[22]^ Differences in lifestyle factors may also account for these outcomes; for example, men are 1.5-times more likely than women to smoke.^[23]^ Smoking is a risk factor for breast cancer metastasis, including bone and lung metastases.^[24,25]^

However, metastatic MEC patients had a lower incidence of lung only metastasis than did metastatic FEC patients; this finding is consistent with that of previous studies.^[7,26]^ The lung-only metastatic pattern in MEC patients was different from that observed in patients with gastric cancer and hepatocellular carcinoma. ^[27,28]^ The reason behind this phenomenon is unknown, which might indicate that many more MEC patients with lung metastasis had simultaneously other distant organ metastasis, and further studies are needed to explain this. The rate of simultaneous bone and lung metastases was similar in both MEC and FEC patients. Single-site metastasis was the most common metastatic pattern in both MEC and FEC patients, followed by 2- and 3-site metastasis; 4-site metastasis was the least common type.

In the present study, there were differences in clinicopathological characteristics and metastatic patterns between metastatic MEC and FEC patients. However, the overall survival rates were similar in both groups and among patients with the most common metastatic sites (the liver, bone, lung, liver, and lung). ^[9]^

Similar findings were reported in other malignant tumors such as breast and colorectal cancers with distant metastases, among others.^[29,30]^ Compared to non-metastatic MEC patients, metastatic MEC patients had poor overall survival (*P* < .001). Multivariable Cox regression revealed that distant organ metastasis was associated with poor prognosis in MEC patients (Table 3). In addition, among all metastatic patterns, simultaneous liver and lung metastases were associated with the poorest prognosis. Overall, the prognosis associated with metastatic sites was poor. In the present study, radiotherapy did not seem to affect outcomes; this finding is in contrast to that of previous studies. However, this study did not account for radiotherapy dose or target due to the lack of data. However, the role of both palliative and definitive radiotherapy in the treatment of metastatic EC is controversial.^[31–35]^ Clinical trials are required to validate the present findings.

### 4.1 Limitations

This study has some limitations. First, this study was based on the SEER database, which is a retrospective database, and we could only obtain information on liver, lung, bone, and brain metastases; data on other metastatic sites were not available. Second, only cases of synchronous metastases were recorded; data on asynchronous metastases were lacking. Third, we could not establish what factors were associated with the differences in metastatic patterns between men and women; further research is required to elucidate the mechanisms of these differences. Fourth, the present study sample was extracted from the United States population; the present findings may not generalize to other populations.

## 5 Conclusion

The present study is the first large-scale report on MEC characteristics and metastasis patterns in EC. The present findings are relevant to clinicians managing patients with EC. Although metastatic patterns may differ between MEC and FEC patients, the prognosis may be similar for both patient groups. Distant organ metastasis in EC patients may be a risk factor for poor outcomes.

## Acknowledgments

We would like to thank the staff members of the National Cancer Institute and the researchers who have been involved with the SEER Program.

## Author contributions

Shengqiang Zhang performed data collection, data analysis, and manuscript writing. Jida Guo, Hongyan Zhang, and Huawei Li performed data collection and data analysis. Mohamed Osman Omar Hassan took part in manuscript writing. Linyou Zhang performed project development. All authors contributed to the article and approved the submitted version.

**Conceptualization:** Shengqiang Zhang.

**Data curation:** Shengqiang Zhang, Jida Guo, Hongyan Zhang, Huawei Li.

**Formal analysis:** Jida Guo, Hongyan Zhang, Huawei Li.

**Investigation:** Shengqiang Zhang, Hongyan Zhang.

**Project administration:** Shengqiang Zhang, Linyou Zhang.

**Supervision:** Linyou Zhang.

**Writing – original draft:** Shengqiang Zhang.

**Writing – review & editing:** Mohamed Osman Omar Osman Omar Hassan.
